# Draft Genome Sequence of Israeli Outbreak-Associated *Vibrio vulnificus* Biotype 3 Clinical Isolate BAA87

**DOI:** 10.1128/genomeA.00032-14

**Published:** 2014-03-20

**Authors:** Kelsey E. Phillips, Matthew J. Schipma, Karla J. F. Satchell

**Affiliations:** a Department of Microbiology-Immunology, Northwestern University, Feinberg School of Medicine, Chicago, Illinois, USA; b Next-Generation Sequencing Core Facility, Northwestern University, Feinberg School of Medicine, Chicago, Illinois, USA

## Abstract

*Vibrio vulnificus* is a seafood-associated pathogen that causes severe wound and intestinal infections. Biotype 3 of *V. vulnificus* emerged in 1996 as the cause of an Israeli outbreak associated with the handling of infected tilapia. Here, we describe the whole-genome sequence of the ATCC biotype 3 clinical isolate BAA87 (CDC9530-96).

## GENOME ANNOUNCEMENT

*Vibrio vulnificus* is a Gram-negative bacterial pathogen found in coastal waters and causes gastroenteritis, primary septicemia, and necrotizing fasciitis in humans. The mortality rates for primary sepsis and wound infection are 50% and 15%, respectively, and the annual incidence of infections is increasing with climate change (1–4). The presence of *V. vulnificus* in oysters and other shellfish within coastal waters poses a large risk to the humans that handle them, and thus the characterization of this pathogen is critical (5). Strains have previously been classified as either biotype 1 (BT1), which is most commonly associated with clinical infections, or biotype 2 (BT2), which causes infections in eels (6–8). However, between 1996 and 1999, an outbreak caused by *V. vulnificus* of the biotype 3 (BT3) lineage resulted in wound infections due to the handling of infected tilapia (9). Biotype 3 strains now cause wound infections in handlers of fish from the seas of Israel, with a death rate of 10% and survivors experiencing severe morbidity (10). Due to the sudden emergence and clonality of the Israeli BT3 strains (11), it is hypothesized that this is a newly emerged pathogen, and thus further investigation into the BT3 genome is pertinent in order to determine how the genomic content may affect disease progression.

In this paper, we describe the draft genome sequence of the clinical isolate *V. vulnificus* strain ATCC BAA87 (CDC9530-96), which was isolated from a patient wound during the original Israel epidemic in 1996. A total of 53.5 ng/μl of genomic DNA was extracted using the Qiagen DNeasy genomic DNA prep for Gram-negative bacterial cultures, according to the manufacturer’s instructions. The DNA library was generated by the Northwestern University Genomics Core Facility and subsequently sequenced using the Life Technologies Ion Torrent PGM technology, specifically the 318 Chip paired with a 400-bp library. The genome was assembled using Newbler (GS *de novo* Assembler version 2.7) from 454 Life Sciences, with 1,022,286 total reads generating 218 contigs from 400-bp trimmed reads. The genome was aligned to *V. vulnificus* BT3 environmental isolate VVyb1(BT3) (12) using Torrent Mapper (TMAP) version 3.4.1. BAA87 has a 97.5% alignment to the reference genome, with an average coverage of 52×. The assembled genome was annotated using the Rapid Annotations using Subsystems Technology (RAST), a SEED-based prokaryotic genome annotation service (13).

### Nucleotide sequence accession numbers.

The whole-genome shotgun project has been deposited at DDBJ/EMBL/GenBank under the accession no. JDSE00000000. The version described in this paper is version JSDE00000000.1.
